# Alarming levels of heat shock proteins 72 and 90α in critically ill children

**DOI:** 10.1186/cc14120

**Published:** 2015-03-16

**Authors:** M Fitrolaki, H Dimitriou, M Venihaki, M Katrinaki, G Briassoulis

**Affiliations:** 1 University Hospital, Heraklion, Greece; 2 University of Crete, Medical School, Heraklion, Greece

## Introduction

Extracellular heat shock proteins (HSP) act as inducers of interleukins (IL) and stimulants for immune cells during systemic inflammatory response syndrome (SIRS). Little is known about the alarming roles of extracellular HSP72 and HSP90α in the acute phase [1] of sepsis (S) or severe sepsis (SS). We determined serum HSP90α, HSP72 and neutrophil CD64 expression, IL-6, IL-8, IL-10, and TNFα in children with S or SS compared with SIRS (brain injury) or healthy children (H).

## Methods

Critically ill children with S (*n *= 16), SS (*n *= 15) or SIRS (*n *= 18) and H (*n *= 21) were enrolled in the study. ELISA was used to evaluate HSPs, chemiluminescence to measure ILs, and flow cytometry to evaluate nCD64 expression (IRB approved).

## Results

Patients in both septic groups had elevated HSP90α (*P *< 0.0001), HSP72 (*P *< 0.05), IL-6 (*P *< 0.0001), IL-8 (*P *< 0.02) and IL-10 (*P *< 0.05) levels compared with H, whereas SS had increased HSP72, IL6 and TNFα compared with SIRS (*P *< 0.05). SIRS patients presented increased HSP90α, IL-6 and IL-8 compared with H (*P *< 0.05). Both HSPs were dramatically increased among nonsurvivors. In a logistic regression model, only HSP90α was independently associated with mortality (*P *< 0.0001). HSP90α related positively (*P *< 0.001) to nCD64, IL-8, IL-10, CRP, PRISM, PELOD, TISS, and LOS and negatively to HDL (*P *< 0.001) and LDL (*P *< 0.02). HSP72 also related negatively to HDL (*P *< 0.001).

## Conclusion

Extracellular HSP72 and HSP90α are alarmingly elevated in critically ill children, especially in severe sepsis. HSP90α levels are independently associated with mortality, related to CD64, IL-8, IL-10, severity of illness, and outcome. Both HSPs are inversely related to the low LDL/low HDL septic metabolic pattern [2].

## Acknowledgements

This research has been co-financed by the European Union (European Social Fund) and Greek national funds through the Operational Program 'Education and Lifelong Learning' of the National Strategic Reference Framework Research Funding Program: THALES. Investing in knowledge society through the European Social Fund.
